# Supplementary material for: Vasoactive Properties of a Cocoa Shell Extract: Mechanism of Action and Effect on Endothelial Dysfunction in Aged Rats
Source: Antioxidants (Basel). 2022 Feb 21;11(2):429. doi: 10.3390/antiox11020429 (PMC8869230; doi:10.3390/antiox11020429)
Supplement: Supplementary file 1 [file antioxidants-11-00429-s001.zip › antioxidants-1531965-supplementary.pdf]

Supplementary Materials

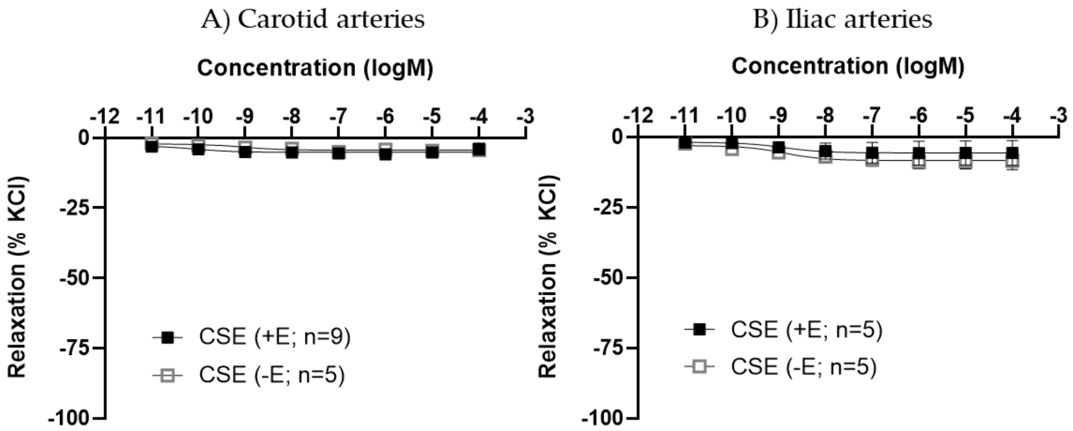

Figure S1. Concentration-response curves to CSE under basal condition in carotid and iliac arteries from female adult rats. Data show the effect in carotid and iliac artery segments with and without endothelium. Relaxation is expressed as a percentage of maximal contraction to NA (10^{-7} M). Data represent the mean ± SEM; n, indicates the number of segments from 4-5 different rats per group; statistical analysis was performed by 2-way ANOVA. ACh, Acetylcholine; CSE, cocoa shell extract; +E, segments with endothelium; -E, segments without endothelium.

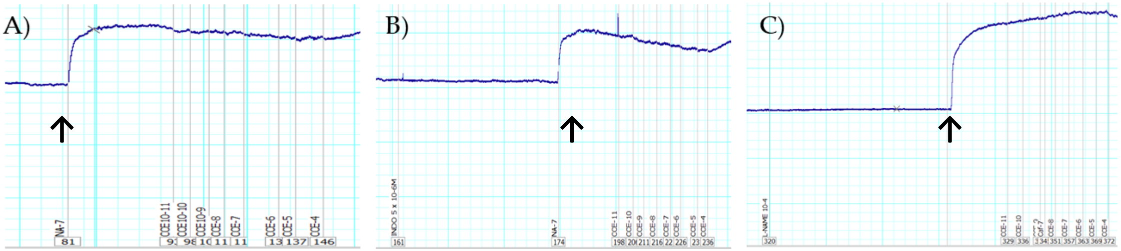

Figure S2. Representative recordings of the effect of indomethacin and L-NAME on CSE relaxations in iliac arteries from adult male rats. Arteries were precontracted with NA (10^{-7} M; black arrow). CSE curve prior to preincubation (A); after preincubation for 20 min with $5{\times}10^{-6}$ M indomethacin (B), and after pre-incubation for 20 minutes with 10^{-4} M L-NAME (C).

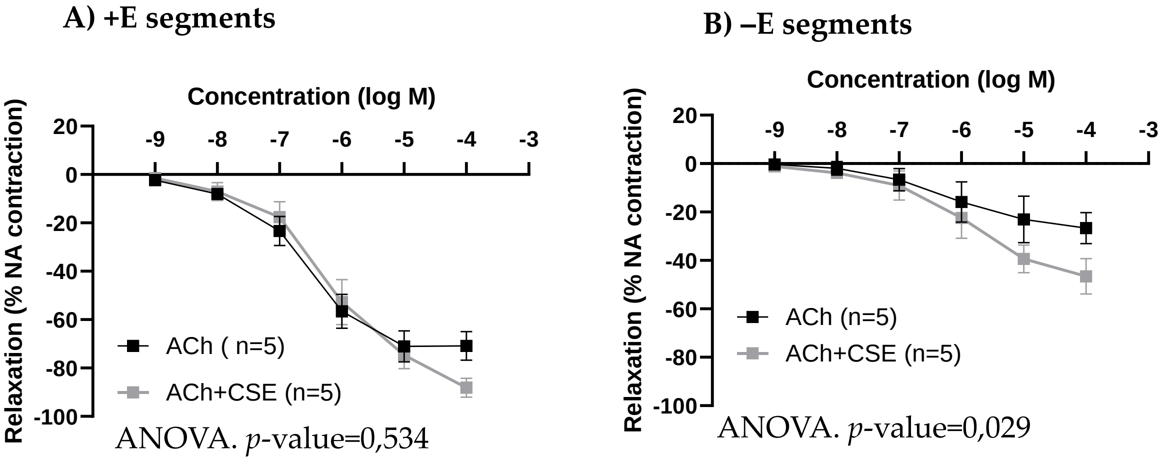

Figure S3. Effect of pre-incubation for 20 min with 10^{-4} M CSE on ACh relaxations in iliac arteries from adult male rats. Relaxation responses were expressed as percentages of maximal contraction with NA (10^{-7} M). Data represent the mean ± SEM; n, indicates the number of segments. Statistical analysis was performed by 2-way-ANOVA, considering concentration and CSE preincubation as factors. The *p*-Value was extracted of the interaction term of the ANOVA; ACh, Acetylcholine; CSE, cocoa shell extract; +E, segments with endothelium; -E, segments without endothelium.

Table S1. Effect of preincubation with CSE, CAF, TH or PCA on NA contractions.

Compound	10^{-7} M NA (g)	10^{-7}M NA +compound (g)	*p*-Value	10^{-6}M NA +compound (g)	*p*-Value
CSE	1.79±0.34	1.77±0.28	0.960	Not tested	
CAF	2.22±0.38	0.96±0.14	0.023	2.08±0,40	0.440
TH	2.21±0.24	2.30±0.22	0.530	Not tested	
PCA	1.95±0.19	2.12±0.24	0.230	Not tested	

Effect of pre-incubation with 10^{-4} M CSE, CAF, TH or PCA on NA contractions in iliac arteries from aged male rats. Contractions are expressed in grams. *p*-value was obtained by paired-Student´s t test; NA, noradrenaline; CSE, cocoa shell extract; TH, theobromine; CAF, caffeine; PCA, protocatechuic acid.

Table S2. Effect of L-NAME on maximal relaxation to CSE in arteries from aged females.

Artery	Max. CSE (%)	Max. CSE + L-NAME (%)	*p*-Value
Iliac (n=4)	-30.6±8.9	+6.4±3.2	0.006
Carotid (n=4)	-40.0±6.9	+5.8±2.0	0.003

Maximum relaxation (Max.) is shown as mean ± SEM. The *p*-Value was extracted by Student's t tests. CSE, cocoa shell extract; n, indicates the number of segments.
